# Systems Biology Will Direct Vascular-Targeted Therapy for Obesity

**DOI:** 10.3389/fphys.2020.00831

**Published:** 2020-07-15

**Authors:** Yingye Fang, Tomasz Kaszuba, P. I. Imoukhuede

**Affiliations:** Imoukhuede Systems Biology Laboratory, Department of Biomedical Engineering, McKelvey School of Engineering, Washington University in St. Louis, St. Louis, MO, United States

**Keywords:** systems biology, obesity, angiogenesis, lymphangiogenesis, adipose tissue vasculature, quantitative flow cytometry, computational modeling, VEGFR

## Abstract

Healthy adipose tissue expansion and metabolism during weight gain require coordinated angiogenesis and lymphangiogenesis. These vascular growth processes rely on the vascular endothelial growth factor (VEGF) family of ligands and receptors (VEGFRs). Several studies have shown that controlling vascular growth by regulating VEGF:VEGFR signaling can be beneficial for treating obesity; however, dysregulated angiogenesis and lymphangiogenesis are associated with several chronic tissue inflammation symptoms, including hypoxia, immune cell accumulation, and fibrosis, leading to obesity-related metabolic disorders. An ideal obesity treatment should minimize adipose tissue expansion and the advent of adverse metabolic consequences, which could be achieved by normalizing VEGF:VEGFR signaling. Toward this goal, a systematic investigation of the interdependency of vascular and metabolic systems in obesity and tools to predict personalized treatment ranges are necessary to improve patient outcomes through vascular-targeted therapies. Systems biology can identify the critical VEGF:VEGFR signaling mechanisms that can be targeted to regress adipose tissue expansion and can predict the metabolic consequences of different vascular-targeted approaches. Establishing a predictive, biologically faithful platform requires appropriate computational models and quantitative tissue-specific data. Here, we discuss the involvement of VEGF:VEGFR signaling in angiogenesis, lymphangiogenesis, adipogenesis, and macrophage specification – key mechanisms that regulate adipose tissue expansion and metabolism. We then provide useful computational approaches for simulating these mechanisms, and detail quantitative techniques for acquiring tissue-specific parameters. Systems biology, through computational models and quantitative data, will enable an accurate representation of obese adipose tissue that can be used to direct the development of vascular-targeted therapies for obesity and associated metabolic disorders.

## Introduction

The prevalence of obesity has tripled since 1975, affecting over 650 million adults worldwide (World Health Organization, 2018). Obesity treatment includes diet and lifestyle changes, physical exercise, and/or surgical procedures (Cao, 2014). However, obesity treatment outcomes are often complicated by accompanying metabolic disorders (Muñoz-Garach et al., 2016; Acharya and Shukla, 2018; Neeland et al., 2018). For instance, metabolically unhealthy obesity is associated with complications such as insulin resistance and type 2 diabetes. Patients with metabolically unhealthy obesity are at a higher risk for chronic disease and death compared to metabolically healthy obese individuals (Durward et al., 2012; Neeland et al., 2018). Alarmingly, the metabolically unhealthy obese phenotype comprises at least 70% of the obese population. Therefore, treating obesity while improving metabolic health is critical for protecting these obese individuals from future health problems (Blüher, 2010; Lemoine et al., 2013; Cao, 2013, 2018; Fukumura et al., 2016; Incio et al., 2018).

The dependency of adipose tissue growth and metabolism on adipose vasculature indicates that vascular-targeted therapies could be used to treat obesity and obesity-associated metabolic disorders (Cao, 2014; Escobedo and Oliver, 2017). Blood and lymphatic vessels formed via angiogenesis and lymphangiogenesis, respectively, are critical for maintaining tissue oxygenation, removing waste products, and regulating adipose tissue expansion and inflammatory responses (Chakraborty et al., 2019). Impaired vasculature can cause tissue hypoxia, leakage of lipids, and chronic inflammation, which are key factors that drive pathological transitions from metabolically healthy to metabolically unhealthy obesity (Corvera and Gealekman, 2014). Restoring vascular health requires a careful balance because upregulation of pro-angiogenic signaling has the downside of accelerating adipose tissue growth during obesity and overexpression of pro-lymphangiogenic factors can increase dietary lipid absorption in the intestinal lymphatics. Therefore, designing a vascular-targeted therapeutic strategy that both reduces adipose tissue mass and also prevents developing adverse metabolic outcomes would require precise control of angiogenic/lymphangiogenic signaling (Nijhawans et al., 2020).

## VEGF:VEGFR Signaling Regulate Angiogenesis, Lymphangiogenesis, Adipogenesis, and Macrophage Specification

**FIGURE 1:**
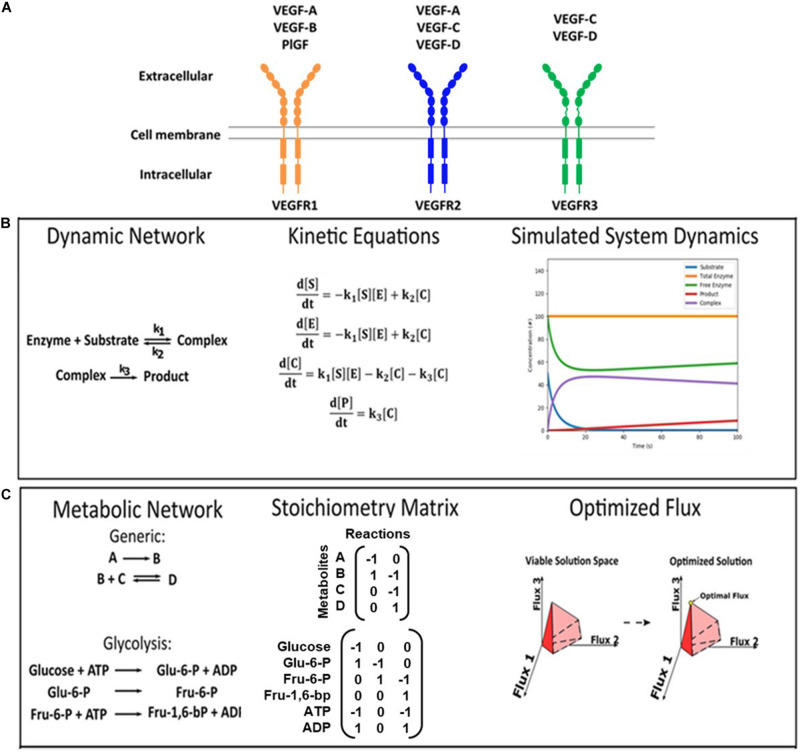
VEGF ligand/receptor interactions and modeling approaches. **(A)** This schematic summarizes the canonical VEGF:VEGFR interactions. VEGF-A binds to both VEGFR1 and VEGFR2. VEGF-B and PlGF exclusively bind to VEGFR1. VEGF-C and VEGF-D bind to both VEGFR2 and VEGFR3. VEGFR2 on vascular endothelial cells primarily induces pro-angiogenic functions, and VEGFR3 on lymphatic endothelial cells primarily induces pro-lymphangiogenic functions. **(B,C)** Formulations of kinetic deterministic and constraint-based models. **(B)** An example of a dynamic enzymatic network was made into kinetic equations, and the output was simulated. **(C)** Examples of inputs for a generic network and glucose metabolism are shown, as well as a visual representation of the output.

Vascular development is primarily regulated by vascular endothelial growth factors (VEGFs) and VEGF receptors (Cao, 2014). VEGFs were initially known as vascular permeability factors that promote tumor vessel permeability (Senger et al., 1983) and, later, they were recognized as vascular growth factors that regulate angiogenesis and lymphangiogenesis. The VEGF family ligands (VEGF-A, -B, -C, -D, and placental growth factor PlGF) selectively bind to three membrane-bound tyrosine kinase receptors, VEGFR1, VEGFR2, and VEGFR3 (Figure 1A). Dimerization of VEGFRs forms either homo- or hetero-dimers, enabling downstream signaling that initiates hallmark angiogenic and lymphangiogenic responses, including cell migration, proliferation, survival, and matrix reorganization (Karkkainen and Petrova, 2000; Ferrara et al., 2003; Tammela et al., 2008; Koch and Claesson-welsh, 2012; Karaman et al., 2018). In this section, we discuss how the VEGF:VEGFR system is involved in obesity, with regards to its role in angiogenesis, lymphangiogenesis, adipogenesis, and macrophage specification, and the current state of development of VEGF/VEGFR-targeted therapies for treating obesity.

### VEGF/VEGFR: Angiogenesis and Lymphangiogenesis

VEGFR1 and VEGFR2 are the primary angiogenic receptors, with different ligand-induced signaling responses (Rahimi, 2006) which makes knowing the distribution of VEGFR1/VEGFR2 on endothelial cells important for predicting angiogenic outcomes. Here, we highlight six functional differences between VEGFR1 and VEGFR2 that underscore this importance: (1) VEGF-A binds to VEGFR1 with ∼10 times stronger affinity than to VEGFR2 (Cunningham et al., 1999; Von Tiedemann and Bilitewski, 2002; Mamer et al., 2017). (2) However, VEGFR1 initiates lower VEGF-A induced proliferation as measured by 3H-thymidine assays, (3) lower migratory activity as seen in Boyden chamber assays, and (4) less actin reorganization as determined by imaging membrane ruffling, when comparing VEGFR1-transfected to VEGFR2-transfected endothelial cells (Waltenberger et al., 1994). (5) Furthermore, VEGFR1 and VEGFR2 regulate endothelial specification in sprouting angiogenesis: high VEGFR2 is preferentially found on tip cells, the leading cells of an angiogenic sprout, while (6) high VEGFR1 is preferentially found on stalk cells, the trailing cells of an angiogenic sprout (Gerhardt et al., 2003; Bentley et al., 2008; Blanco and Gerhardt, 2013). Hence, VEGFR2 has been established as the major pro-angiogenic VEGFR (Apte et al., 2019) and VEGFR1 is believed to modulate angiogenic signaling and under certain conditions competitively inhibit VEGFR2 signaling. Indeed, the VEGFR1 modulatory versus inhibitory duality lies in its ability to prevent excessive vascular growth in embryos while promoting angiogenesis in cancer, ischemic, and adipose tissues (Carmeliet et al., 2001; Ho and Fong, 2015; Robciuc et al., 2016; Lacal and Graziani, 2018). The dependence of angiogenic outcomes on VEGFR competition makes quantifying the VEGFR distribution a crucial step for predicting angiogenic behaviors in adipose tissue.

It is also prudent to consider VEGFR heterodimers in adipose tissue angiogenesis, as both VEGFR1 and VEGFR3 can heterodimerize with VEGFR2 on vascular endothelial cells, affecting the angiogenic response. Whether VEGFRs preferentially form homo- or hetero-dimeric complexes is an unresolved matter; however, simulations estimate that 10–50% of VEGFRs are heterodimeric. Furthermore, the VEGFR density is predicted to regulate homo- vs. hetero-dimerization: a ratio of 10:1 VEGFR1:R2 or 10:1 VEGFR2:R1 inhibits homodimerization of the less-abundant receptor (R2/R2 or R1/R1, respectively) (Mac Gabhann and Popel, 2007). Functionally, VEGFR1/R2 heterodimerization is significant because it increases VEGFR1 phosphorylation and leads to significantly stronger ligand-induced migration than either VEGFR2/R2 or VEGFR1/R1 homodimerization (Huang et al., 2001; Cudmore et al., 2012). VEGFR2/R3 heterodimers on vascular endothelial cells also result in stronger ligand-induced angiogenic sprouting than when the heterodimerization was prevented by VEGFR3 antibodies (Nilsson et al., 2010). Overall, VEGFR heterodimerization is a prevalent phenomenon that can upregulate endothelial cell migration and angiogenic sprouting relative to homodimers; thus, accounting for heterodimerization can lead to improved predictions of VEGF-induced cell responses.

Another key vascular process is lymphangiogenesis, which is primarily driven by VEGFR3 activation on lymphatic endothelial cells. VEGFR3 is necessary for lymphangiogenesis as it activates PROX1, the master transcription factor regulating both differentiation of lymphatic endothelial cells from their progenitors and subsequent maintenance of their lymphatic identity (Jha et al., 2018). VEGFR2 is also indispensable for lymphangiogenesis, as inhibition of VEGFR2/R3 heterodimers significantly reduces ligand-induced VEGFR3 phosphorylation (Alam et al., 2004) and completely blocks VEGF-C-induced lymphatic sprouting (Nilsson et al., 2010). Additionally, the VEGFR2 antibody (DC101) prevents the onset of lymphatic vessel growth in adult mice (Goldman et al., 2007). As we have discussed, the relative amounts of VEGFR2 and VEGFR3 will affect the formation of VEGFR2/R3 heterodimers on lymphatic endothelial cells; therefore, quantitative characterization of the distributions of VEGFRs on lymphatic endothelial cells is similarly required to accurately predict the lymphangiogenic responses of adipose tissue.

### VEGF/VEGFR: Isoforms and Signaling Crosstalk

Many VEGF isoforms initiate differential angiogenic and lymphangiogenic responses, which increases the complexity of VEGF:VEGFR signaling. VEGF-C and -D are matured by proteolytic processing, which is necessary for their interaction with VEGFR2 (Joukov et al., 1997; Achen et al., 1998; Shin et al., 2008). Unlike VEGF-C (-D) isoforms, the isoforms of VEGF-A, -B, and PlGF are derived from alternative mRNA splicing; these isoforms have been reviewed in greater detail elsewhere (Weddell and Imoukhuede, 2018; Bowler and Oltean, 2019). For brevity, we discuss only the splice variants of VEGF-A. VEGF-A_165_ is the most abundant isoform; other known VEGF-A isoforms include VEGF-A_111_, VEGF-A_121_, VEGF-A_145_, VEGF-A_148_, VEGF-A_18__3_, VEGF-A_189_, and VEGF-A_206_ (Bowler and Oltean, 2019). These VEGF-A isoforms differ in their binding affinities with the VEGFR co-receptors and the extracellular matrix (ECM) components, differentially regulating VEGFR binding and activation (Park et al., 1993; Robinson and Stringer, 2001; Jakobsson et al., 2006; Sarrazin et al., 2011; Nieminen et al., 2014; Sarabipour and Mac Gabhann, 2018; King and Hristova, 2019). Therefore, anti-VEGF studies conducted using pan-VEGF antibodies without distinguishing the various isoforms could be potentially less accurate.

There are also VEGF-A isoforms termed VEGF-A_(xxx)a_ and VEGF-A_(xxx)b_ which are generated via exon 8 splicing and designated as pro-angiogenic and anti-angiogenic, respectively (Kopp et al., 2006). These VEGF-A_(xxx)a/b_ splice variants are discussed by several reviews (Ladomery et al., 2007; Biselli-Chicote et al., 2012; Hilmi et al., 2012). Here, we highlight three VEGF-A_165__*b*_ properties that are implicated in obesity: (1) VEGFR binding: VEGF-A_165b_ competes with VEGF-A_165a_ for VEGFR binding in pathological conditions (Woolard et al., 2004; Mamer and Wittenkeller, 2016a, b; Clegg and Mac Gabhann, 2017). Thus, adipose tissue angiogenesis can be inhibited by increasing the VEGF-A_165b_:VEGFR2 binding. (2) VEGFR activation: VEGF-A_165b_ induces only weak VEGFR phosphorylation and downstream signaling in ischemia (Clegg et al., 2017; Ganta et al., 2017); indeed, markedly lowered angiogenic activity has been observed in VEGF-A_165b_-high visceral adipose tissue compared to VEGF-A_165b_-low subcutaneous adipose tissue (Ngo et al., 2014). (3) Systemic VEGF-A_165b_ upregulation: obese patients show systemic upregulation of VEGF-A_165b_ compared to lean patients, which is reduced after significant weight loss following bariatric surgery (Ngo et al., 2014). Thus, systemic upregulation of VEGF-A_165b_ might be a prognostic marker for weight loss outcomes. However, it remains unclear whether this VEGF-A_165b_ upregulation in the systemic circulation and visceral adipose tissue is a compensatory mechanism to reduce the excessive growth of adipose tissue or a pathological mechanism leading to vascular disease.

A recent study of VEGF-A_(xxx)b_ mRNA *in vivo* further complicates this story: VEGF-A_(xxx)b_ mRNA was undetectable in RNA-seq data from approximately 7,000 samples from 50 tissues (Bridgett et al., 2017). They suggested that studies of VEGF-A_(xxx)b_ may be confounded by variants that contain both “a” and “b” C-terminal sequences, termed VEGF-A_*x*_. Such VEGF-A_*x*_ variants would be detected by VEGF-A_(xxx)b_ antibody (Eswarappa et al., 2014) which was used in the previous study on VEGF-A_(xxx)b_ in obesity (Ngo et al., 2014). VEGF-A_*x*_ also has putative anti-angiogenic effects. Thus, additional studies are needed to determine whether the systemic VEGF-A_165b_ upregulation in obese patients is, in fact, VEGF-A_165x_ upregulation and to determine the functional consequences of these anti-angiogenic isoforms in adipose tissue.

VEGFRs also exist in soluble forms that competitively bind and sequester VEGFs from the membrane-bound VEGFRs. For instance, soluble VEGFR1 is a high-affinity receptor for VEGF-A and thereby downregulates the pro-angiogenic VEGFR2 signaling (Shibuya, 2011). Soluble VEGFR3 can similarly inhibit lymphangiogenesis by preventing VEGF-C/D from binding membrane-bound VEGFR3 (Mäkinen et al., 2001). The anti-VEGF effect of soluble VEGFRs could be a critical factor that alters the outcomes of a VEGF:VEGFR signaling system.

VEGF:VEGFR signaling is further complicated by non-canonical signaling involving the platelet-derived growth factor family (PDGFs). The angiogenic signaling canon describes uni-family interactions: VEGF:VEGFR signaling promoting healthy vascular formation (Tammela et al., 2008; Sina et al., 2011; Simons, 2012; Dellinger et al., 2013; Simons et al., 2016) and PDGF:PDGFR signaling on perivascular cells supporting blood vessel function (Kazlauskas, 2017). Recent discoveries of PDGF binding to VEGFR2 (Mamer et al., 2017; Kazlauskas, 2018), VEGF-A:PDGFR signaling (Ball et al., 2007; Pennock and Kazlauskas, 2012; Pennock et al., 2014), and VEGF regulation of PDGFRs (Chen et al., 2015) reveal that the canonical, uni-family paradigm insufficiently describes vascular development. It would be worthwhile to study whether PDGF contributes to VEGFR signaling in obesity, as (1) PDGF is secreted by macrophages, pre-adipocytes, and adipocytes; (2) the concentration of PDGF ligands in serum and the expression of PDGF mRNA in adipose tissue cells were both shown to increase by ∼1.5× in obese mice; and (3) anti-PDGF antibody reduced endothelial tube formation compared to untreated control (Pang et al., 2008). As such, non-canonical signaling should also be considered when elucidating angiogenic mechanisms in obesity.

### VEGF/VEGFRs in Adipogenesis and Obesity

Adipose tissue angiogenesis is driven by either local hypoxia or endocrine signaling (Silvia, 2014). Under hypoxia, rapid adipose tissue expansion activates hypoxia inducible factor (HIF)-regulated genes, inducing the transcription, translation, and secretion of pro-angiogenic factors like VEGFs and PDGFs in both adipocytes and endothelial cells. In the endocrine mechanism, circulating insulin, growth factors (e.g., VEGFs and FGFs), and nutrients (e.g., amino acids and glucose) activate adipocyte transcriptional pathways (Um et al., 2006), among which mTOR activation is the pivotal switch that triggers local adipose HIF-1 and VEGF production (Jia et al., 2015). Indeed, multiple signaling pathways help coordinate these adipose tissue angiogenesis mechanisms, as detailed in several reviews (Cao, 2013; Escudero et al., 2017; Korybalska, 2018) despite the many angiogenic regulators, VEGF:VEGFR signaling is accepted as the primary adipose angiogenic pathway (Sung et al., 2013). In white adipose tissue, upregulation of VEGF-A:VEGFR2 signaling can promote tissue vascularization, increasing oxygen supply and subsequently enhancing energy consumption (di Somma et al., 2020). In addition, VEGF-A causes vascular fenestration, one of the structural bases that cause vascular permeability (Kamba et al., 2006). VEGF-A:VEGFR2 signaling promotes adipose vascular permeability, which allows the release of free fatty acids from adipose tissue to the circulation when systemic energy is low during fasting (Asterholm et al., 2012). Taken together, VEGF:VEGFR signaling plays an important role in controlling adipose tissue function and energy metabolism through the modulation of the adipose vasculature.

Angiogenesis also supports adipose tissue expansion by providing a niche for preadipocyte recruitment and differentiation (Tang et al., 2008; Volz et al., 2016). Preadipocytes recruited to the vascular niche differentiate into mature adipocytes via adipogenesis, assisting existing non-proliferative adipocytes in storing glucose, lipids, and cholesterol. Newly formed adipocytes cluster around active angiogenic sprouts, as observed via live adipose tissue imaging (Nishimura et al., 2007). This intimate spatial association enables paracrine signaling that increases both adipogenesis and angiogenesis. Microvascular endothelial cells enhance adipogenesis by secreting insulin-like growth factors and fibroblast growth factors (FGF) (Volz et al., 2016). FGF-1 was shown to facilitate differentiation of human preadipocytes that was initiated by PPARγ (Hutley et al., 2004), which is the master transcriptional regulator of adipogenesis (Sarjeant and Stephens, 2012; Pellegrinelli et al., 2016). Reciprocally, preadipocytes and mature adipocytes secrete various pro-angiogenic factors, including VEGF-A and FGF-2, which permit further angiogenesis and adipose tissue expansion (Volz et al., 2016; Jiang et al., 2017). This endothelial-adipocyte crosstalk has been reviewed in further detail elsewhere (Volz et al., 2016). Altering either angiogenesis or adipogenesis will inevitably affect this paracrine loop, resulting in changes to both processes.

Impaired lymphangiogenesis is also implicated in the progression of obesity. The peripheral lymphatic vessels are required for lipid transport and immune cell clearance in adipose tissue. Impaired lymphatic vasculature, commonly seen in obesity, can leak lipid-containing lymph into peripheral tissue, inducing adipogenesis, subcutaneous fat deposition, and weight gain in mice (Escobedo et al., 2016; Escobedo and Oliver, 2017). Inactivating mutations in VEGFR3 in Chy mice induce lymphatic vessel leakiness and cause abnormal adipose tissue accumulation adjacent to these impaired vessels. Treating the Chy mice with an adenovirus encoding VEGF-C can stimulate lymphatic growth, suggesting VEGFR3 activation with an excess of its ligand is sufficient to overcome the lymphatic impairment (Karkkainen et al., 2001). Because VEGF-C can also stimulate vascular permeability via binding VEGFR2, this study further suggested that VEGFR3-specific ligand (VEGF-C156S) is a more attractive choice for therapeutically activating VEGFR3 and stimulating lymphangiogenesis. Thus, upregulating VEGFR3 activation could be useful for restoring lymphatic vessel integrity and reducing adipose tissue expansion. In addition, obese mice with leaky lymphatic vessels exhibited blunted immune cell trafficking toward lymph nodes, higher peak inflammatory responses, and delayed clearance of inflammatory responses when compared to lean mice with healthy vessels (Savetsky et al., 2015). The same study showed that VEGF-C (a VEGFR3 ligand) upregulated lymphangiogenesis, improved immune cell clearance, and decreased tissue inflammation, suggesting a therapeutic approach for alleviating adipose tissue inflammation in obesity. Altogether, repairing the impaired lymphatics can help reduce adipose tissue expansion and inflammation in obesity, which can be done by normalizing VEGFR3 activation.

Lacteals, the specialized lymphatic vessels adjacent to the small intestines, are important for absorbing digested dietary lipids and conveying the lipids to the circulation (Harvey, 2008; Jiang et al., 2019). Emerging evidence suggests that reducing lacteal permeability can inhibit lipid absorption during high-fat diet, additional work is needed to develop a therapy targeting the lacteals (Shew et al., 2018; Cifarelli and Eichmann, 2019). Lacteal permeability can be reduced via upregulating VEGF-A:VEGFR2 signaling, which transforms discontinuous button-like cell junctions to continuous zipper-like junctions, limiting lipid entry into the lacteals (Zhang et al., 2018). Thus, administration of VEGF-A or VEGFR1 inhibitors are promising therapeutic options for reducing lacteal permeability and lipid uptake. A further understanding of the lacteal zippering mechanism holds promise for the identification of therapeutic targets for obesity treatment.

Established *in vivo* obesity models should be used in further studies investigating the molecular and cellular mechanisms that link angiogenesis/lymphangiogenesis, adipogenesis, and related metabolic outcomes. High-fat diet (HFD)-fed mice are widely used models for obesity research because they exhibit (1) increased triglyceride storage via adipocyte hypertrophy, (2) increased overall body weight (Jo et al., 2009) and (3) metabolic dysfunction, such as insulin resistance, i.e., impaired control of blood glucose via insulin (Samuel et al., 2010). Future efforts should take advantage of these *in vivo* models to collect quantitative data on the molecular and cellular changes caused by VEGF/VEGFR-targeted therapies; such efforts will help identify key signaling mechanisms that affect obesity progression and response to therapies.

### VEGF/VEGFR: Macrophage Function in Adipose Tissue

Pro-inflammatory macrophage accumulation is considered a major factor that perpetuates chronic inflammation in obese adipose tissue. HFD-induced obesity increased the number of pro-inflammatory M1 macrophages by 65-fold while increasing anti-inflammatory M2 macrophages by only six-fold (Fujisaka et al., 2009). M1 macrophages secrete pro-inflammatory molecules, like IL-6 and TNF-alpha, and are associated with an impaired ability to control blood glucose by insulin, also known as insulin resistance. Conversely, M2 macrophages are more prevalent in lean adipose tissue, and they maintain insulin levels by releasing anti-inflammatory cytokines (Mills et al., 2000; Lumeng et al., 2007; Castoldi et al., 2016). Increasing the number of M2 relative to M1 macrophages may improve metabolic outcomes of obese patients, as an increased M1:M2 ratio is associated with metabolically unhealthy obesity (Fujisaka et al., 2009).

Macrophages are VEGFR-expressing cells and the ratio of M1/M2 macrophages in obese adipose tissue can be normalized by modulating the VEGF/VEGFR expression. For instance, VEGF-A antibody can reduce the number of M2 macrophages in the hypoxic adipose tissue tip region, which is the region of active adipose outgrowth characterized by a dense vascular network; these M2 macrophages are important for tissue outgrowth as they secrete matrix metalloproteinases (MMPs) (Cho et al., 2007). The evidence that VEGF-A regulates M2 abundance is further substantiated by a later study, which showed that overexpression of VEGF-A can drive the composition of macrophages in adipose tissue toward low-M1/high-M2, protecting mice from diet-induced insulin resistance (Elias et al., 2012). Meanwhile, VEGFR1 signaling in macrophages was also found to favor M2 macrophage accumulation, both during wound healing in diabetic mice (Okizaki et al., 2016) and during tumor growth in obese mice (Incio et al., 2016). Evidence from an *in vitro* study suggests that this increase in M2 macrophages could be a result of M1 macrophages differentiating into M2 macrophages, rather than M2 recruitment. This study found that VEGFR1 mRNA was highly expressed in M1 macrophages but only at low levels in M2 macrophages and that VEGF-A:VEGFR1 signaling stimulates M1 recruitment and subsequent differentiation of M1 macrophages into M2 macrophages (Wheeler et al., 2018). Altogether, VEGF-A and VEGFR1 promote the accumulation of anti-inflammatory M2 macrophages, which provides a useful therapeutic strategy to lower inflammation in obese adipose tissue. Future studies should investigate the differential VEGF:VEGFR signaling mechanisms underlying the recruitment and differentiation of M1 and M2 macrophages, such that their responses can be reliably predicted.

Besides the role of M1 and M2 macrophages in inflammation, they also differentially regulate the initiation and continuation of angiogenesis (Corliss et al., 2017). M2 macrophages secrete MMP-9, which degrades the extracellular matrix to initiate vascular sprouting into the adipose tissue tip region (Cho et al., 2007). Both M1 and M2 macrophages secrete pro-angiogenic growth factors, such as VEGFs and PDGFs, and it has been shown that the conditioned medium of either M1 or M2 macrophages promoted endothelial tube formation (Jetten et al., 2014). However, coculture of endothelial cells with M1 macrophages exhibited significantly less tube formation than M2-endothelial cell co-culture (Jetten et al., 2014). Tube formation is necessary for the continuation and stabilization of angiogenic sprouting; the coculture results indicate that the M1-endothelial cell contact is anti-angiogenic, although the M1 paracrine signaling is pro-angiogenic. The mechanisms for this anti-angiogenic behavior remain unclear; however, they could be related to macrophage-endothelial interactions seen in tumor metastasis, where macrophages disrupt endothelial tight junctions to facilitate cell extravasation (Kim et al., 2019). It is also possible that the high VEGFR1 on M1 macrophages sequesters VEGF-A from acting on endothelial cells in coculture. Future studies are needed to investigate how VEGFR signaling is regulated among M1, M2, and endothelial cells present in the same microenvironment.

Although macrophages are conventionally characterized by M1 and M2 subtypes, other classification schemes based on macrophage functions are useful for identifying the roles of macrophages in specific pathologies (Mosser and Edwards, 2008). As we have discussed, VEGF:VEGFR signaling in macrophages affects their recruitment and differentiation into different subtypes, but it is not clear whether these subtypes are strictly M1 or M2 macrophages. Moreover, it is notable that VEGFR-expressing cell subpopulations can express drastically different amounts of VEGFR proteins and generate heterogenous VEGF-induced cell responses, even though they are identified as the same cell type (Weddell and Imoukhuede, 2014). Thus, a VEGFR-based macrophage classification may help identify the subpopulations and functions of macrophages involved in adipose tissue inflammation, angiogenesis, and lymphangiogenesis. Future data on VEGFR distributions in adipose tissue macrophages are necessary.

### Vascular-Targeted Therapies for Obesity

Emerging evidence has shown that adipose vasculature is a promising anti-obesity target (Daquinag et al., 2011; Cao, 2010, 2016). Indeed, since angiogenesis is required for adipose tissue expansion, researchers have already begun testing anti-angiogenic treatments to reduce adipose tissue mass and prevent obesity-associated metabolic disorders. A promising vascular-targeted drug, adipotide (a prohibitin-targeting peptide), has previously shown rapid weight loss in mice by specifically inducing apoptosis of adipose endothelial cells (Kolonin et al., 2004), however, its first human trial was discontinued in 2019 due to unspecified reasons.

Anti-VEGF and anti-VEGFR treatments have also largely been shown to be beneficial in treating obesity in pre-clinical obesity models (Cao, 2014; Leong-Poi, 2014). However, as we have discussed, angiogenesis and adipogenesis are concomitant processes, so there is a delicate balance that needs to be maintained. It is a reasonable concern that anti-angiogenic treatment could reduce adipogenesis to below what is necessary to relieve the metabolic stress of excessively hypertrophic adipocytes (Cao, 2007; Rutkowski et al., 2009; Lemoine et al., 2012; Haczeyni et al., 2018). Indeed, inhibiting angiogenesis can block necessary adipogenesis and two studies have independently shown that: (1) administration of VEGF-A inhibitors not only decreases angiogenic sprouting but also reduces adipogenesis in obese mice (Nishimura et al., 2007); (2) VEGFR2 antibody inhibits both vascular growth and adipogenesis in murine preadipocyte implants, similar to the effect of gene suppression of PPARγ (Fukumura et al., 2003). Thus, anti-angiogenic treatments might worsen metabolic dysfunction in metabolically unhealthy obese patients, who already show impaired angiogenesis and adipogenesis (McLaughlin et al., 2007; Kusminski et al., 2016; Longo et al., 2019). Next, we focus on the consistent and discrepant outcomes from two anti-VEGFR and two anti-VEGF pre-clinical obesity studies; along with other relevant evidence, we discuss the considerations for future anti-VEGFR/VEGF therapies for obesity treatment.

Anti-VEGFR treatments have shown varied effects on weight loss and metabolic enhancement in HFD-fed and chow-fed models. Systemic anti-VEGFR2 treatment significantly slowed weight gain after 6 weeks of HFD, though no weight suppression was shown in the first 6 weeks (Tam et al., 2009). In comparison, systemic anti-VEGFR1 treatment showed no effect on body weight throughout the HFD treatment (Tam et al., 2009). Although targeting VEGFR1 did not suppress weight gain in obese mice, systemic anti-VEGFR1 treatment resulted in positive metabolic outcomes in chow-fed mice, which exhibited increased adipose vascular density, reduced adipocyte size, and enhanced metabolic energy expenditure (i.e., increased non-shivering thermogenic capacity). These features were observed over a 10-day anti-VEGFR1 treatment but not observed with anti-VEGFR2 treatment (Seki et al., 2018). Moreover, targeted gene deletion of endothelial VEGFR1 also led to a healthier metabolic profile, including lowered blood levels of free fatty acids, glycerol, cholesterol, fasting serum levels of glucose, and serum levels of insulin in HFD-fed mice (Seki et al., 2018). Altogether, both anti-VEGFR1 and anti-VEGFR2 have shown benefit in either weight loss or metabolic improvement; however, it is unclear what variables may affect individual responses to treatments.

Improved metabolic function was also observed in two pre-clinical anti-VEGF studies, although there were discrepancies in the weight loss outcomes (Honek et al., 2014; Wu et al., 2014). One study showed anti-VEGF treatment decreased serum glucose levels in HFD mice, suggesting that anti-VEGF treatment can mitigate HFD-induced insulin resistance (Honek et al., 2014). The other study showed that anti-VEGF treatment prevented HFD-induced hepatic glucose production but did not alter muscle glucose production, suggesting that anti-VEGF treatment can reduce serum glucose by inhibiting its production in the liver (Wu et al., 2014). However, Wu et al. (2014) did not observe a change in adipocyte size or mouse weight while Honek et al. (2014) observed reductions in both obesity measures. Honek et al. (2014) showed that anti-VEGF-induced adipocyte size reduction was greater in older mice, so the different outcomes regarding adipocyte size reduction may be explained by the age differences between the Wu and Honek studies (2-month vs. 7-month old, respectively). Future studies should also precisely control the dosage and duration of anti-VEGF treatment because prolonged and high-potency anti-VEGF is also known to trigger hypoxia and adverse systemic metabolic changes (Cao, 2013; Usui-Ouchi and Friedlander, 2019).

VEGFR3-targeted therapeutics for obesity have not yet been significantly explored, although the VEGFR3 activation must be maintained or upregulated to prevent lymphatic vessel leakiness, excess adipocyte hypertrophy, and insulin resistance (Cifarelli and Eichmann, 2019). A recent mouse model indicated that increasing the VEGFR3 on the lymphatic endothelium can restore impaired lymphangiogenesis in HFD-fed hyperglycemic conditions (Wu et al., 2018). However, VEGF-C/D:VEGFR3 signaling is also chemotactic, inducing macrophage accumulation and metabolic dysfunction. For instance, dermal production of VEGF-C gene induced pro-inflammatory macrophage accumulation and worsened metabolic parameters such as insulin resistance in transgenic obese mice (Karaman et al., 2015, 2016). Interestingly, adipose-specific overexpression of VEGF-D gene increased lymphatic vessel density in obese adipose tissue and prevented insulin resistance under HFD (Chakraborty et al., 2019). Taken together, upregulating VEGFR3 signaling is a potential therapeutic strategy to repair lymphangiogenesis in obesity and adipose-targeted administration of VEGFR3 ligands might be more beneficial than systemic approaches. Systemic and adipose-targeted administration of VEGF-C (-D) or other VEGFR3 agonists remain to be tested in obese models.

While vascular-targeted therapies are promising, their efficacy in treating obesity remains unclear in humans. Many factors should be considered in future studies targeting VEGF/VEGFR, including age, treatment duration, as well as the routes of administration (systemic or adipose-targeted). For example, systemic administration of VEGFR2 antibody could undesirably increase lacteal permeability, leading to higher lipid uptake from the small intestines to the lymphatics (Zhang et al., 2018). Other factors that we have discussed in this review, such as the abundance of membrane VEGFRs, the distributions of VEGFRs in different cell types, and VEGF splice variants, could also confound the therapeutic outcomes. We believe establishing biologically faithful platforms that predict how the VEGF:VEGFR signaling system regulates vascular density and metabolism in humans through systems biology could offer a mechanistic approach to develop effective therapeutics for obesity and obesity-related metabolic disorders.

## Systems Biology Provides a Platform for Investigating Vascular Signaling and Metabolism in Adipose Tissue

Systems biology offers a mechanistic understanding of complex biological networks through mathematical and computational modeling (Mac Gabhann and Popel, 2009; Weddell and Imoukhuede, 2018; Papin and Mac Gabhann, 2019). Systems biology approaches have led to discoveries of therapeutic biomarkers and have helped design effective vascular-targeted therapies (Weddell and Imoukhuede, 2018). We highlight two highly used systems biology modeling approaches that would be useful for designing better vascular-targeted therapies to treat obesity: (1) mass-action kinetic modeling and (2) constraint-based modeling. We also provide an overview of quantitative techniques for acquiring biologically faithful parameters for accurate modeling.

### Mass-Action Kinetic Modeling

Simulations of VEGF/VEGFR targeted therapy in human patients can be made at the protein level using mass-action kinetic modeling. This approach reconstructs a reaction network using ordinary differential equations (ODEs) (Figure 1B), which requires quantitative parameters on concentrations of ligands and receptors and kinetic reaction rates to simulate behaviors such as protein binding, trafficking, and phosphorylation over time (Loskot et al., 2019). Mass-action kinetic models of VEGF:VEGFR signaling are well-established in the field of angiogenesis (Weddell and Imoukhuede, 2018). These validated models provide a framework that can be easily adapted to represent obesity with adipose tissue-specific parameters. Indeed, several of these models are available in Systems Biology Markup Language, a file format that can be readily used by several software, including MATLAB’s SimBiology and Wolfram’s SystemModeler (SBML, 2016; Software Guide/SBML Software Matrix – SBML.caltech.edu). Reactions and parameters can be modified to test new model implementations in these software. There is also a well-developed community that can critique VEGF:VEGFR model results, which improves the quality of the model and leads to greater credibility in its findings (10 Simple Rules with Conformance Rubric | Interagency Modeling and Analysis Group). Therefore, adipose tissue-specific VEGF:VEGFR models would be in line with the field, while offering a platform for predicting the efficacy of VEGF/VEGFR targeted therapies in obese patients.

The model developed by Finley et al. (2015) provides a useful framework to simulate VEGF/VEGFR targeted therapy in obese adipose tissue. It is the most recent in a series of models investigating the pharmacokinetics of anti-VEGF therapy and was validated with published clinical measurements (Finley et al., 2011, 2013, 2015). The base model is an early benchmark model consisting of 40 ODEs that described receptor trafficking, VEGF secretion and transport, and association and dissociation of free VEGF-A_121_ and VEGF-A_165_ isoforms to membrane VEGFRs, NRP1, and the ECM (Stefanini et al., 2008). Finley et al. added drug, molecular, and lymphatic mechanisms that were relevant to breast cancer, expanding the base model to 161 ODEs. For simplicity, we highlight three features that would be relevant to an obesity adaptation: (1) interactions with the anti-VEGF drug Aflibercept, which could be directly used to model anti-VEGF therapy in obesity; (2) VEGF:soluble VEGFR1 binding, which sequesters VEGF from interacting with membrane VEGFRs, could downregulate membrane VEGFR activation and subsequent angiogenesis/adipogenesis in adipose tissue. Additional predictions of soluble VEGFR1 dynamics can be found in earlier computational studies (Wu et al., 2009, 2010); and (3) lymphatic drainage of macromolecules from tissue compartments into the blood compartment, which can model changes in lymphatic vessel integrity seen in obese and diabetic conditions (Savetsky et al., 2014; Scallan et al., 2015) and can be extended to clarify whether normalizing VEGFR3 signaling can restore lymphatic functions. Overall, adapting the Finley et al. (2015) model with adipose tissue-specific parameters (e.g., VEGFR concentrations on adipocytes and adipose stromal-vascular cells) will allow accurate prediction of the pharmacokinetics of anti-VEGF therapies in obese patients.

However, many other signaling mechanisms could affect the efficacy of vascular-targeted therapies, like the competition between pro- and anti-angiogenic VEGF-A isoforms, cross-family PDGF-VEGFR2 signaling, and paracrine signaling between the endothelium and adipocytes/macrophages, as we detailed earlier. To more accurately predict how obese adipose tissue responds to vascular-targeted therapies, future models should integrate the ODEs that have been developed to model: the impact of VEGFR heterogeneity on anti-VEGF efficacy (Weddell and Imoukhuede, 2014) the differential VEGF signal transduction by VEGFR homo- and hetero-dimers (Mac Gabhann and Popel, 2007; Mamer et al., 2019b), the significance of VEGF-A_165b_ in ischemic conditions (Chu et al., 2016; Clegg et al., 2017), the effect of cross-family PDGF-VEGFR binding on VEGFR occupancy (Mamer et al., 2017), the VEGFR mechanisms inducing macrophage migration (Weddell et al., 2018), the recruitment of macrophages to the lymphatic endothelium (Bianchi et al., 2015), and the pro- and anti-inflammatory signaling of adipose tissue macrophages (Díaz et al., 2009). Modeling these molecules, interactions, and cell responses would allow us to determine which mechanisms in adipose tissue could be targeted to normalize VEGF:VEGFR signaling in obesity.

### Constraint-Based Modeling

There is also a significant need to understand and improve how vascular-targeted therapies affect metabolism. Mass-action kinetic modeling has only been applied by a few laboratories for modeling metabolic networks, as it is a challenge to acquire the reaction rates of all the interactions in these large and complex systems (Rizzi et al., 1997; Chassagnole et al., 2002; Yugi et al., 2005; Tran et al., 2008; Jamshidi and Palsson, 2010). In this regard, constraint-based modeling (CBM) has been an effective alternative to simulate a metabolic network and identify its regulatory mechanisms and dysfunctional pathways (Mardinoglu et al., 2013b; Bordbar et al., 2014). CBM does this via three steps: (1) Describing each pathway via stoichiometric equations: Figure 1C exemplifies one glycolysis reaction in stoichiometric form; (2) Incorporating experimental data on metabolite concentrations: unlike mass-action kinetic modeling, CBM requires only concentration measurements. Metabolite concentrations in pathological, healthy, and treatment conditions can be obtained from primary experiments, mining literature, or databases like the Human Protein Atlas [available from^1^ Uhlen et al. (2015)] and NCBI’s Gene Expression Omnibus (Edgar, 2002; Barrett et al., 2019). These concentrations are used as the initial conditions of the model; and (3) Simulating and analyzing the pathway: these final steps reveal the rates, also known as “fluxes”, through each pathway. The stoichiometric equations built in Step 1 dictate how the model proceeds from the initial conditions set in Step 2, and comparing the flux profiles of different conditions in Step 3 will indicate which pathways are used at a greater or lesser flux (Orth et al., 2010).

In order to make a CBM for studying metabolic changes in obese adipose tissue in response to vascular-targeted therapies, we highlight a model that is available in SBML format and can be used as a benchmark model for studying pathogenesis and therapeutic strategies in adipocyte metabolism (Mardinoglu et al., 2013a)^2^. All known metabolic reactions of the human adipocyte were curated from prior models, experimental evidence, and database mining. This model ultimately involved 1809 genes which regulated 6160 reactions between 4550 metabolites. This model can be adapted to mechanistically explore the transcriptional pathways that connect VEGF:VEGFR signaling and adipocyte metabolism. For instance, this model can be used to reconstruct the VEGF-activated mitochondrial thermogenic activity and the browning of white adipocytes, and identify the key mitochondrial genes that regulate the VEGF expression in such processes (di Somma et al., 2020). In addition, metabolites were separated by organelle (e.g., cytosol, mitochondria, extracellular space) to obtain a compartment-specific reconstruction. While several other CBM approaches have investigated metabolism in physiological adipogenesis, obesity, and diabetes (Si et al., 2007; Coskun et al., 2010; Bordbar et al., 2011; Väremo et al., 2013; Montastier et al., 2015) we believe this model would provide the best benchmark for researchers to use for investigating the response to vascular-targeted therapies due to its thorough reconstruction of adipocyte metabolism and inclusion of VEGFR-related pathways.

The findings from the Mardinoglu et al. (2013a) model also provide an example of the information that can be gained through CBM (Mardinoglu et al., 2013a). Using various proteomics data from lean and obese adipose tissue (e.g., uptake/secretion rates of non-esterified fatty acids (NEFAs), triacylglycerol (TAG), and glucose), they simulated the dynamics of lipid droplet formation and acetyl-CoA production, the central metabolite of the mitochondria. They found that lean patients showed larger fluctuations in the size of lipid droplets than obese patients, as determined by comparing the flux through a reaction that synthesized lipid droplets from its constituent components (TAGs, NEFAs, cholesterol, phospholipids, etc.). They also found that obese patients exhibited lower acetyl-CoA production, which indicated potential mitochondrial dysfunction. They then extended the former investigation to include gene regulation using proteomic and genomic data from a clinical study on obese and non-obese individuals (SOS Sib Pair study), which allowed them to study global metabolic changes occurring in obesity. They predicted downregulated flux through all mitochondrial metabolic pathways in the obese patients of this study, further supporting the link between obesity and mitochondrial dysfunction (Bournat and Brown, 2010). Additionally, they identified three upregulated metabolites that could serve as biomarkers for obesity: (1) androsterone, a steroid hormone that may increase adipose tissue deposition (O’Reilly et al., 2014), (2) heparan sulfate proteoglycan (HSPG) degradation products, where HSPGs regulate the transport of lipoprotein lipase across endothelial cells to adipocytes (Saxena et al., 1991), and (3) ganglioside GM2, a molecule associated with insulin signaling in adipocytes (Tanabe et al., 2009). These findings demonstrated that CBM can identify specific changes in metabolic pathways and biomarkers, which can be extended to discover the potential adverse effects of vascular-targeted therapies on metabolic health and biomarkers for predicting patients’ metabolic outcomes.

### Techniques for Acquiring Quantitative Parameters for Predictive Models

Quantitative data are necessary to construct and validate computational models that are predictive of vascular signaling in adipose tissue so that they can be used to improve vascular-targeted therapies for obesity. Binding kinetics parameters, such as VEGF:VEGFR association and dissociation rates, are necessary for mass-action kinetic modeling. Binding kinetics can be acquired with affinity assays, commonly using radiolabeling and surface plasmon resonance (SPR) (Mamer et al., 2019a). In addition, protein concentrations, such as serum VEGF concentrations and membrane VEGFR concentrations, are required for both CBM and mass-action kinetic modeling. Soluble protein concentrations (e.g., VEGF-A, soluble VEGFRs) are commonly obtained using quantitative enzyme-linked immunosorbent assays (ELISAs) and radiolabeling assays as well. However, there is a lack of a standardized method for obtaining quantitative data on membrane VEGFR concentrations, which are especially important for predicting VEGF/VEGFR signaling outcomes.

Quantitative flow cytometry (qFlow) is an emerging quantitative standard for measuring proteins on the plasma membrane (Chen et al., 2017; Wu and Finley, 2017). qFlow employs calibration beads to translate the mean fluorescence intensity values to fluorophore molecules per cell (Imoukhuede and Popel, 2011; Chen et al., 2017). VEGFR quantification has applied phycoerythrin (PE)-conjugated VEGFR antibodies to cells (Chen et al., 2018), and PE calibration beads are used to convert the PE signal intensity to the number of PE molecules per cell. The number of PE molecules per cell equals the number of PE-conjugated receptors per cell, due to the 1:1 protein/fluorophore ratio. Thus, applying qFlow with PE-conjugated antibodies allows absolute quantification of membrane VEGFR levels.

Furthermore, cell-by-cell analysis of qFlow data can unmask population heterogeneity when specific subpopulations depart from the “mean” behavior (Altschuler and Wu, 2010; Weddell and Imoukhuede, 2014). To identify and characterize such subpopulations in VEGFR-expressing cells (e.g., endothelial cells and macrophages), the qFlow measurements should be analyzed in 2 steps: (1) fitting the cell-by-cell VEGFR distribution to multiple log-normal distributions using mixture modeling and (2) optimizing the fitting by applying Bayesian information criterion (BIC) to identify the number of subpopulations without overfitting (Chen et al., 2018). BIC is preferred over Akaike information criterion (AIC) because AIC can report more false-positive subpopulations in qFlow data, increasing mixture model complexity with small sample sizes (Vrieze, 2012). Therefore, cell-by-cell qFlow analysis and mixture modeling should be used for identifying cell subpopulations exhibiting distinct receptor expression and VEGFR-mediated cell responses in adipose tissue.

qFlow has successfully measured membrane receptor levels on tumor xenograft-derived cells (Imoukhuede and Popel, 2014; Chen et al., 2018), primary mouse skeletal muscle endothelial cells under normal (Imoukhuede and Popel, 2012), and ischemic (Imoukhuede et al., 2013) conditions, and several *in vitro* cell lines (Chen et al., 2015; Weddell et al., 2018; Chen and Imoukhuede, 2019). To summarize some of the key qFlow findings, human umbilical vein endothelial cells (HUVECs) present 1800 ± 200 VEGFR1/cell, 4900 ± 400 VEGFR2/cell, and 2800 ± 400 VEGFR3/cell. These VEGFR concentrations on the membrane are regulated by VEGFs. A 24-h VEGF-A treatment increases the concentration of membrane VEGFR1 while decreasing membrane VEGFR2 on HUVECs; likewise, a 24-h VEGF-C treatment decreases the concentration of membrane VEGFR3 on in-vitro human lymphatic endothelial cells (Imoukhuede and Popel, 2011). The membrane VEGFR distributions have also been examined in the endothelium from healthy mouse skeletal muscle and tumor xenograft models, where endothelial cells stably present low membrane VEGFR2 (<1700 VEGFR2/cell) across normal and tumor xenografts (Imoukhuede and Popel, 2012, 2014; Chen et al., 2018) despite VEGF-A:VEGFR2 binding being the primary pro-angiogenic signaling pathway (Olsson et al., 2006). On the other hand, endothelial cells from breast tumor xenografts overexpress membrane VEGFR1 relative to HUVECs, presenting an average of 15,000 membrane VEGFR1/cell. Although it is still unclear why VEGFR1 overexpression is associated with pathologically active angiogenesis, qFlow measurements have enabled predictions of the outcomes of VEGFR1 overexpression in pathological angiogenesis and cell migration (Weddell and Imoukhuede, 2014, 2018; Mamer et al., 2017; Weddell et al., 2018). For example, computational modeling has predicted that tumor endothelial cells overexpressing membrane VEGFR1 contribute to the therapeutic resistance to anti-VEGF-A treatment because the VEGF-A ligands captured by membrane VEGFR1 can dissociate to increase free VEGF-A levels in tumors, counteracting the anti-VEGF-A treatment (Weddell and Imoukhuede, 2014). This model also predicted that the subpopulation of tumor endothelial cells expressing >35,000 VEGFR1/cell is a potential marker of therapeutic resistance to the anti-VEGF treatment (Weddell and Imoukhuede, 2014). This prediction was further supported by the clinical observation that VEGFR1 overexpression in tumor tissue was correlated with decreased overall survival in patients treated with the anti-VEGF agent bevacizumab (Weickhardt et al., 2015). This prediction was possible because of data from qFlow measurements of VEGFR levels on breast tumor xenograft endothelial cells. Thus, computational models can yield clinically relevant findings when coupled with qFlow data.

**FIGURE 2:**
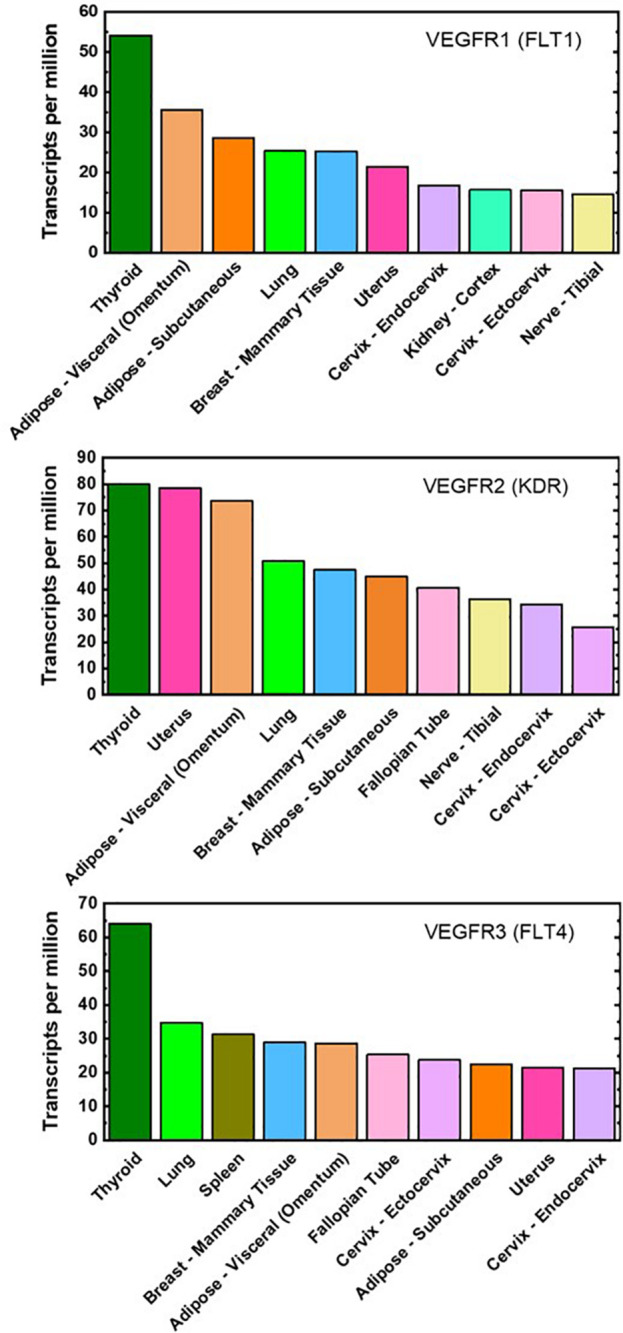
Top ten VEGFR mRNA-expressing tissues. Gene expression of VEGFR1, VEGFR2, and VEGFR3 are ranked highly in both visceral and subcutaneous adipose tissues (light- and dark-orange bar, respectively). These plots are reproduced with median gene-level of VEGFRs and include the top 10 tissues representing the highest VEGFR gene expression. The full database of 53 examined tissues can be accessed at https://gtexportal. org/home/.

To date, there are minimal proteomic data on VEGFR concentrations on adipose-derived cells, even though adipose tissue is one of the highest VEGFR gene-expressing tissues (Figure 2). qFlow can be used to establish the VEGFR distribution in non-obese and obese adipose tissue and characterize adipose tissue cell heterogeneity through cell-by-cell analysis and mixture modeling. qFlow can also address the unmet need for a repository, similar to the GTEx portal (a genomic database), of quantitative protein concentrations in various tissues, particularly adipose tissue. Such a database would enable researchers across labs to contribute and acquire quantitative proteomic data to build systems biology models.

## Systems Biology Enables Future Vascular-Targeted Therapy for Obesity

The predictive power of computational modeling paired with quantitative data should be used to design and direct vascular-targeted therapies for obesity. With the established quantitative techniques (ELISA, SPR, radiolabeling) and literature data, the VEGF:VEGFR kinetics and VEGF concentrations in lean, obese, and treated adipose tissue can be acquired. However, there are minimal proteomic data on VEGFR concentrations on adipose-derived cells, even though adipose tissue is one of the highest VEGFR gene-expressing tissues. qFlow should be used to acquire membrane-bound VEGFR concentrations in adipose tissue; furthermore, cell-by-cell analysis and mixture modeling will be particularly helpful for identifying functionally distinct cell subpopulations within adipose tissue endothelial cells and macrophages.

We have reviewed benchmark models that will provide the basis for future efforts to predict the effects of vascular-targeted therapies on obesity and metabolic responses. These models predict VEGF:VEGFR signaling outcomes and metabolic network regulation via mass-action kinetic modeling and CBM, respectively; mass-action kinetic modeling can be used to design effective vascular-targeted therapeutic strategies, and CBM can identify metabolic pathways that are significantly affected by vascular-targeted therapies. Systems biology, through future quantitative data and computational models, will enable a better understanding of obesity-associated vascular dysregulation and advance vascular-targeted therapies for obesity and its associated metabolic disorders.

## Data Availability Statement

The original contributions presented in the study are included in the article, further inquiries can be directed to the corresponding author.

## Author Contributions

YF led the manuscript preparation. TK contributed to the computational modeling review and contributed critical discussion on this work. YF, TK, and PI prepared the manuscript. All authors contributed to the article and approved the submitted version.

## Conflict of Interest

The authors declare that the research was conducted in the absence of any commercial or financial relationships that could be construed as a potential conflict of interest.

## Footnotes

1 http://www.proteinatlas.org

2 metabolicatlas.org
